# *Pljushtchiaargoi* sp. n., a new geometrid moth from the Western Tien Shan Mountains (Lepidoptera, Geometridae, Larentiinae)

**DOI:** 10.3897/BDJ.10.e82353

**Published:** 2022-03-29

**Authors:** Jaan Viidalepp, Aare Lindt, Olavi Kurina

**Affiliations:** 1 Estonian University of Life Sciences, Institute of Agricultural and Environmental Sciences, Tartu, Estonia Estonian University of Life Sciences, Institute of Agricultural and Environmental Sciences Tartu Estonia; 2 Estonian Museum of Natural History, Tallinn, Estonia Estonian Museum of Natural History Tallinn Estonia

**Keywords:** climate change, new species, taxonomy, Uzbekistan

## Abstract

**Background:**

This paper focuses on the morphological description and illustration of the wing pattern and genitalia structures of the known species of the genus *Pljushtchia*. The possibility of co-evolution of *Pljushtchia* moths and fruit tree forests of Tien Shan is discussed. The maple tree is supposed to have evolved in the Ili River valley in NW China and in Kazakhstan. *Malussieversii*, the wild apple tree, is distributed in Miocene nemoral forest belt to Europe in the West an to western North America in the East. The last remnants of fruit tree forests are now localised in biodiversity hotspots in China and in Middle Asian mountains.

**New information:**

This paper provides a description of a new species of geometrid moth, *Pljushtchiaargoi* sp. n. As the ancestral *Malussieversii* has diverged in *Malussilvestris* in Europe, *Malusturkestanica* in Kyrgyzstan and *Malushissarica* in Tajikistan, a co-divergence of geometrid moths and their food plants is possible. We found *Chlorissaarkitensis* Viidalepp in the Chatkal area, Tien Shan, its sister species *Chlorissatalvei* Viidalepp in Hissar and Pamirs and *Chlorissapretiosaria* Staudinger in Transcaucasus. *Pljushtchiaprima* is associated with a biodiversity hotspot in Tajikistan and *Pljushtchiaargoi* with another biodiversity hotspot in southern Tien Shan.

## Introduction

The genus *Pljushtchia* was described for *P.prima* from the southern slope of Hissar Mts. in Tajikistan (Viidalepp and Kostjuk 2005). The autumnal moths from a remote area are poorly discussed in scientific literature (Parsons et al. 1999, Viidalepp 2011). The purpose of the current review is to describe a new species of the larentiine genus *Pljushtchia* Viidalepp & Kostjuk found in Uzbekistan.

## Materials and methods

**Figure 1.**
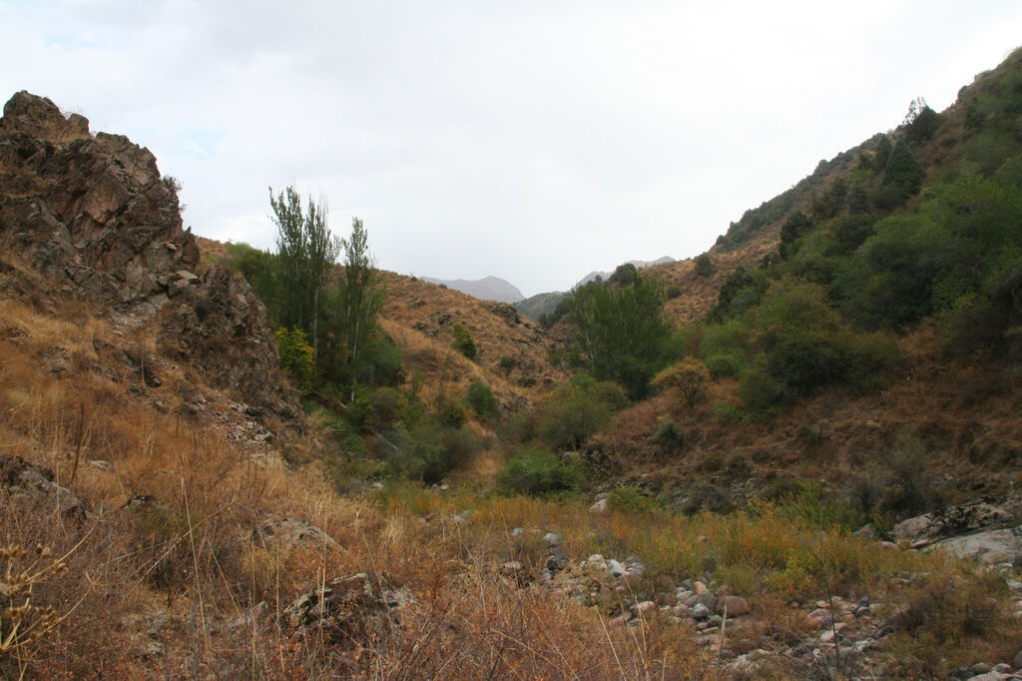
Sampling site landscape in Chatkal NR.

One of the authors (OK) and Mr. A. Selin (Tallinn, Estonia) from the Estonian Lepidopterists' Society collected the material during their entomological expedition in Uzbekistan, on a southern flank of the Chatkal Range of the Western Tien Shan Mountain system. The collecting methods included light trapping and selective sampling near light traps. The light trapping site was at the main building of the Chatkal National Reserve (IUCN category IV) at about 1100 m elevation. The lamps were placed within bush and shrub vegetation, with steppe slopes behind (Fig. 1.)

The Chatkal NR covers about 3500 km^2^ of the forest and alpine biota on the southern slopes of the West Tien Shan range (Zhumanova et al. 2021). The steppe and mountain steppe pastures on lowland and lower elevations around the National Reserve are over-grazed and the grazing stresses the Reserve.

Palpi, antennae, legs and details of the venation of wings were measured on mounted specimens using an ocular micrometer and binocular microscopes, under 40× magnification. The genital slides of males and females were treated using established procedures (Hardwick 1950), inspected in glycerol, embedded in Euparal and, thereafter, photographed from the ventral view. Moths were photographed prior to investigation of the genital structures using a Canon 300D digital camera, while the genital slides were photographed by a Leica DFC295 camera attached to a Leica S6D stereomicroscope. The obtained photographs were augmented using Adobe Photoshop Elements v. 7 in order to clarify their resolution.

## Taxon treatments

### Pljushtchia

Viidalepp & Kostjuk, 2005

532D37D4-E443-59D6-A3E6-3FF33A744DE5

Pljushtchia Pljushtchia prima Viidalepp & Kostjuk, 2005

#### Diagnosis

The genus was diagnosed using the autapomorphic characters of the type species as unipectinate antennae in male and serrate antennae in female. It was identified as a cidariine genus by the venation of fore- and hind-wings and by the presence of a pair of lateral appendages to the juxta, defined as the labides. The association of *Pljushtchia* with *Thera* Stephens, *Protothera* Viidalepp and *Heterothera* Inoue was supported using a cladistic analysis (Viidalepp and Kostjuk 2005). This analysis revealed the existence of four monophyletic groups of genera within the tribe Cidariini. *Thera* and allied genera appeared as the crown group of Cidariini, characterised by the reduction of the posterior or anterior apophyses in females; the valve costa projecting distally or dorsally in male genitalia; the valve sacculus tip projecting beyond the ventral margin of valva in male genitalia and the modification or the absence of the signum in female genitalia. *Pljushtchia* differs from *Thera* in more slender wings, especially in the fore-margin of hind-wing being longer than the hind margin of the forewing.

The *Thera* group of genera has the hind-wing discal vein twice angulate, sharing this character with its sister-group (containing of *Lampropteryx* Stephens, *Nebula* Bruand and others), which is otherwise diagnosed by the presence of bipartite labides or labides provided with blade-like hamuli.

### Pljushtchia prima

Viidalepp & Kostjuk, 2005

FEEBFC7A-1429-53C6-BBCE-545ABADCA9B1

#### Materials

- **Type status:** Holotype. **Occurrence:** catalogNumber: IZBE0085519; recordedBy: Igor Plyushch; individualCount: 1; sex: male; preparations: pinned/gen. prep. #2942; **Taxon:** scientificName: *Pljushtchiaprima* Viidalepp & Kostjuk, 2005; genus: Pljushtchia; specificEpithet: *prima*; scientificNameAuthorship: Viidalepp & Kostjuk, 2005; **Location:** continent: Eurasia; country: Tajikistan; locality: Gissarskiy khr., ushch. Kondara; decimalLatitude: 38.8166; decimalLongitude: 68.8333; **Identification:** identifiedBy: Viidalepp & Kostjuk; **Event:** samplingProtocol: Light trap; year: 1979; month: October; day: 3; **Record Level:** type: Physical Object; institutionCode: EMY; collectionCode: IZBE; basisOfRecord: Preserved Specimen
- **Type status:** Paratype. **Occurrence:** catalogNumber: IZBE0085520; recordedBy: Igor Plyushch; individualCount: 1; sex: female; preparations: pinned/gen. prep. #3915; **Taxon:** scientificName: *Pljushtchiaprima* Viidalepp & Kostjuk, 2005; genus: Pljushtchia; specificEpithet: *prima*; scientificNameAuthorship: Viidalepp & Kostjuk, 2005; **Location:** continent: Eurasia; country: Tajikistan; locality: Gissarskiy khr., ushch. Kondara; decimalLatitude: 38.8166; decimalLongitude: 68.8333; **Identification:** identifiedBy: Viidalepp & Kostjuk; **Event:** samplingProtocol: Light trap; year: 1979; month: September; day: 30; **Record Level:** type: Physical Object; institutionCode: EMY; collectionCode: IZBE; basisOfRecord: Preserved Specimen

#### Diagnosis

*Pljushtchiaprima* was described by Viidalepp and Kostjuk (2005) as medium-sized (wingspan 21-26 mm, females on average smaller than males), brownish light grey with forewing postmediane, antemediane and basale fasciae blackish and edged by whitish scales. Discal spots small, black on a pale blotch each. Hind-wing brownish grey, lighter than forewing, postmediane fascia grey, inconspicuous, cell-spot minute. Male genitalia, as illustrated by Viidalepp and Kostjuk (2005), Fig. 3, with tegumen longer than vinculum, the latter compressed laterally and projecting medially. Valva without medial or costal ornamentation, distal-ventral part characteristically emarginated. Juxta broad and short, plate shaped, with short labides on its lateral lobes. Aedeagus shorter than valva, with two sets of needle-shaped cornuti on vesica. Female genitalia small, membranous.

#### Biology

The species is autumnal, monovoltine, confined to remnants of wild fruit tree forests and orchards. Degtjareva (1973) and Degtjareva (1981)have studied the lepidopterous fauna of these fruit tree and broad-leaved forests on the southern slope of Hissar Mountains and in Karategin Mts.

### Pljushtchia argoi

Viidalepp, Lindt & Kurina sp. n.

12E88D56-3CBD-5588-A49D-ADDFBDC2B360

C376B7B5-BD5A-4715-ABB3-11255D2F7D35

#### Materials

- **Type status:** Holotype. **Occurrence:** catalogNumber: IZBE0136561; recordedBy: Olavi Kurina; sex: male; preparations: pinned/ gen. prep #8899; **Taxon:** scientificName: *Pljushtchiaargoi* Viidalepp, Lindt & Kurina, 2022; genus: Pljushtchia; specificEpithet: argoi; scientificNameAuthorship: Viidalepp, Lindt & Kurina, 2022; **Location:** continent: Eurasia; country: Uzbekistan; locality: Chatkal NR; cordon at Bashkuzil-saj; verbatimElevation: 1114 m; decimalLatitude: 41.1752; decimalLongitude: 69.8202; **Identification:** identifiedBy: Viidalepp J; **Event:** samplingProtocol: light trap; year: 2009; month: September; day: 20; **Record Level:** type: PhysicalObject; institutionCode: EMY; collectionCode: IZBE; basisOfRecord: PreservedSpecimen
- **Type status:** Paratype. **Occurrence:** catalogNumber: IZBE0136562; recordedBy: Olavi Kurina; sex: female; preparations: pinned; **Taxon:** scientificName: *Pljushtchiaargoi* Viidalepp, Lindt & Kurina, 2022; genus: Pljushtchia; specificEpithet: argoi; scientificNameAuthorship: Viidalepp, Lindt & Kurina, 2022; **Location:** continent: Eurasia; country: Uzbekistan; locality: Chatkal NR; cordon at Bashkuzil-saj; verbatimElevation: 1114 m; decimalLatitude: 41.1752; decimalLongitude: 69.8202; **Identification:** identifiedBy: Viidalepp J; **Event:** samplingProtocol: light trap; year: 2009; month: September; day: 20; **Record Level:** type: PhysicalObject; institutionCode: EMY; collectionCode: IZBE; basisOfRecord: PreservedSpecimen
- **Type status:** Paratype. **Occurrence:** catalogNumber: IZBE0136563; recordedBy: Olavi Kurina; sex: female; preparations: pinned; **Taxon:** scientificName: *Pljushtchiaargoi* Viidalepp, Lindt & Kurina, 2022; genus: Pljushtchia; specificEpithet: argoi; scientificNameAuthorship: Viidalepp, Lindt & Kurina, 2022; **Location:** continent: Eurasia; country: Uzbekistan; locality: Chatkal NR; cordon at Bashkuzil-saj; verbatimElevation: 1114 m; decimalLatitude: 41.1752; decimalLongitude: 69.8202; **Identification:** identifiedBy: Viidalepp J; **Event:** samplingProtocol: light trap; year: 2009; month: September; day: 20; **Record Level:** type: PhysicalObject; institutionCode: EMY; collectionCode: IZBE; basisOfRecord: PreservedSpecimen
- **Type status:** Paratype. **Occurrence:** catalogNumber: IZBE0136564; recordedBy: Olavi Kurina; sex: female; preparations: pinned; **Taxon:** scientificName: *Pljushtchiaargoi* Viidalepp, Lindt & Kurina, 2022; genus: Pljushtchia; specificEpithet: argoi; scientificNameAuthorship: Viidalepp, Lindt & Kurina, 2022; **Location:** continent: Eurasia; country: Uzbekistan; locality: Chatkal NR; cordon at Bashkuzil-saj; verbatimElevation: 1114 m; decimalLatitude: 41.1752; decimalLongitude: 69.8202; **Identification:** identifiedBy: Viidalepp J; **Event:** samplingProtocol: light trap; year: 2009; month: September; day: 20; **Record Level:** type: PhysicalObject; institutionCode: EMY; collectionCode: IZBE; basisOfRecord: PreservedSpecimen
- **Type status:** Paratype. **Occurrence:** catalogNumber: IZBE0136565; recordedBy: Olavi Kurina; sex: female; preparations: pinned; **Taxon:** scientificName: *Pljushtchiaargoi* Viidalepp, Lindt & Kurina, 2022; genus: Pljushtchia; specificEpithet: argoi; scientificNameAuthorship: Viidalepp, Lindt & Kurina, 2022; **Location:** continent: Eurasia; country: Uzbekistan; locality: Chatkal NR; cordon at Bashkuzil-saj; verbatimElevation: 1114 m; decimalLatitude: 41.1752; decimalLongitude: 69.8202; **Identification:** identifiedBy: Viidalepp J; **Event:** samplingProtocol: light trap; year: 2009; month: September; day: 20; **Record Level:** type: PhysicalObject; institutionCode: EMY; collectionCode: IZBE; basisOfRecord: PreservedSpecimen
- **Type status:** Paratype. **Occurrence:** catalogNumber: IZBE0136566; recordedBy: Olavi Kurina; sex: female; preparations: pinned; **Taxon:** scientificName: *Pljushtchiaargoi* Viidalepp, Lindt & Kurina, 2022; genus: Pljushtchia; specificEpithet: argoi; scientificNameAuthorship: Viidalepp, Lindt & Kurina, 2022; **Location:** continent: Eurasia; country: Uzbekistan; locality: Chatkal NR; cordon at Bashkuzil-saj; verbatimElevation: 1114 m; decimalLatitude: 41.1752; decimalLongitude: 69.8202; **Identification:** identifiedBy: Viidalepp J; **Event:** samplingProtocol: light trap; year: 2009; month: September; day: 20; **Record Level:** type: PhysicalObject; institutionCode: EMY; collectionCode: IZBE; basisOfRecord: PreservedSpecimen
- **Type status:** Paratype. **Occurrence:** catalogNumber: IZBE0136567; recordedBy: Olavi Kurina; sex: female; preparations: pinned; **Taxon:** scientificName: *Pljushtchiaargoi* Viidalepp, Lindt & Kurina, 2022; genus: Pljushtchia; specificEpithet: argoi; scientificNameAuthorship: Viidalepp, Lindt & Kurina, 2022; **Location:** continent: Eurasia; country: Uzbekistan; locality: Chatkal NR; cordon at Bashkuzil-saj; verbatimElevation: 1114 m; decimalLatitude: 41.1752; decimalLongitude: 69.8202; **Identification:** identifiedBy: Viidalepp J; **Event:** samplingProtocol: light trap; year: 2009; month: September; day: 20; **Record Level:** type: PhysicalObject; institutionCode: EMY; collectionCode: IZBE; basisOfRecord: PreservedSpecimen
- **Type status:** Paratype. **Occurrence:** catalogNumber: IZBE0136568; recordedBy: Olavi Kurina; sex: female; preparations: pinned; **Taxon:** scientificName: *Pljushtchiaargoi* Viidalepp, Lindt & Kurina, 2022; genus: Pljushtchia; specificEpithet: argoi; scientificNameAuthorship: Viidalepp, Lindt & Kurina, 2022; **Location:** continent: Eurasia; country: Uzbekistan; locality: Chatkal NR; cordon at Bashkuzil-saj; verbatimElevation: 1114 m; decimalLatitude: 41.1752; decimalLongitude: 69.8202; **Identification:** identifiedBy: Viidalepp J; **Event:** samplingProtocol: light trap; year: 2009; month: September; day: 19; **Record Level:** type: PhysicalObject; institutionCode: EMY; collectionCode: IZBE; basisOfRecord: PreservedSpecimen
- **Type status:** Paratype. **Occurrence:** recordedBy: Allan Selin; sex: 1 male, 3 females; preparations: pinned; **Taxon:** scientificName: *Pljushtchiaargoi* Viidalepp, Lindt & Kurina, 2022; genus: Pljushtchia; specificEpithet: argoi; scientificNameAuthorship: Viidalepp, Lindt & Kurina, 2022; **Location:** continent: Eurasia; country: Uzbekistan; locality: Chatkal NR; cordon at Bashkuzil-saj; verbatimElevation: 1114 m; decimalLatitude: 41.1752; decimalLongitude: 69.8202; **Identification:** identifiedBy: Viidalepp J; **Event:** samplingProtocol: light trap; year: 2009; month: September; day: 20; **Record Level:** type: PhysicalObject; collectionCode: Collection A. Lindt; basisOfRecord: PreservedSpecimen
- **Type status:** Paratype. **Occurrence:** recordedBy: Allan Selin; sex: 7 males, 12 females; preparations: pinned; **Taxon:** scientificName: *Pljushtchiaargoi* Viidalepp, Lindt & Kurina, 2022; genus: Pljushtchia; specificEpithet: argoi; scientificNameAuthorship: Viidalepp, Lindt & Kurina, 2022; **Location:** continent: Eurasia; country: Uzbekistan; locality: Chatkal NR; cordon at Bashkuzil-saj; verbatimElevation: 1114 m; decimalLatitude: 41.1752; decimalLongitude: 69.8202; **Identification:** identifiedBy: Viidalepp J; **Event:** samplingProtocol: light trap; year: 2009; month: September; day: 20; **Record Level:** type: PhysicalObject; collectionCode: Collection A. Selin; basisOfRecord: PreservedSpecimen

#### Description

**Figure 2.**
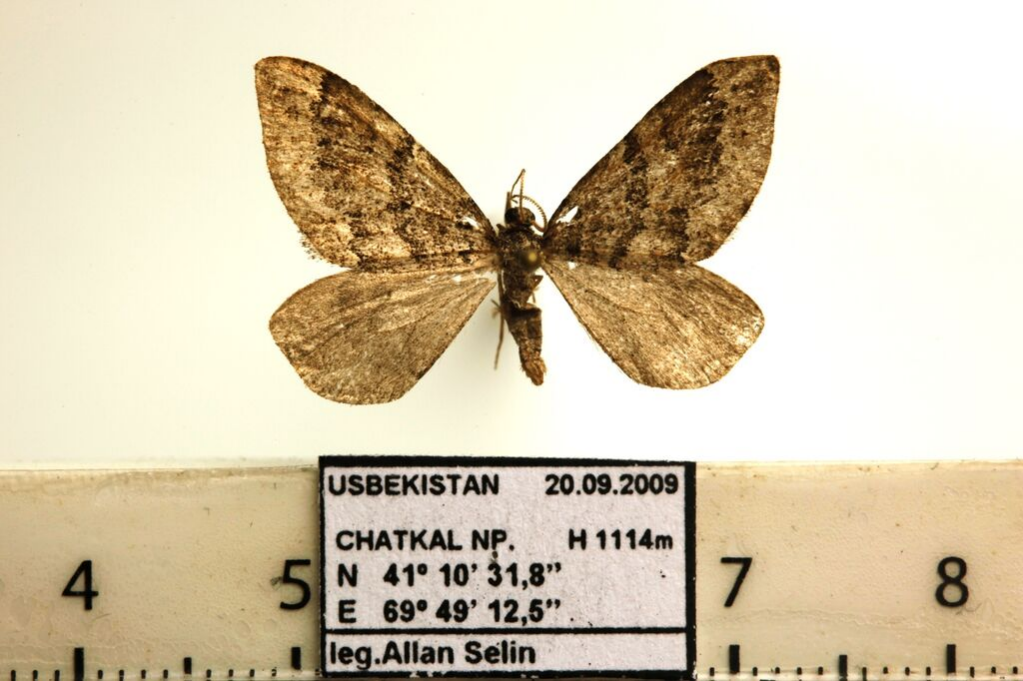
*Pljushtchiaargoi* sp. n. male paratype.

**Figure 3.**
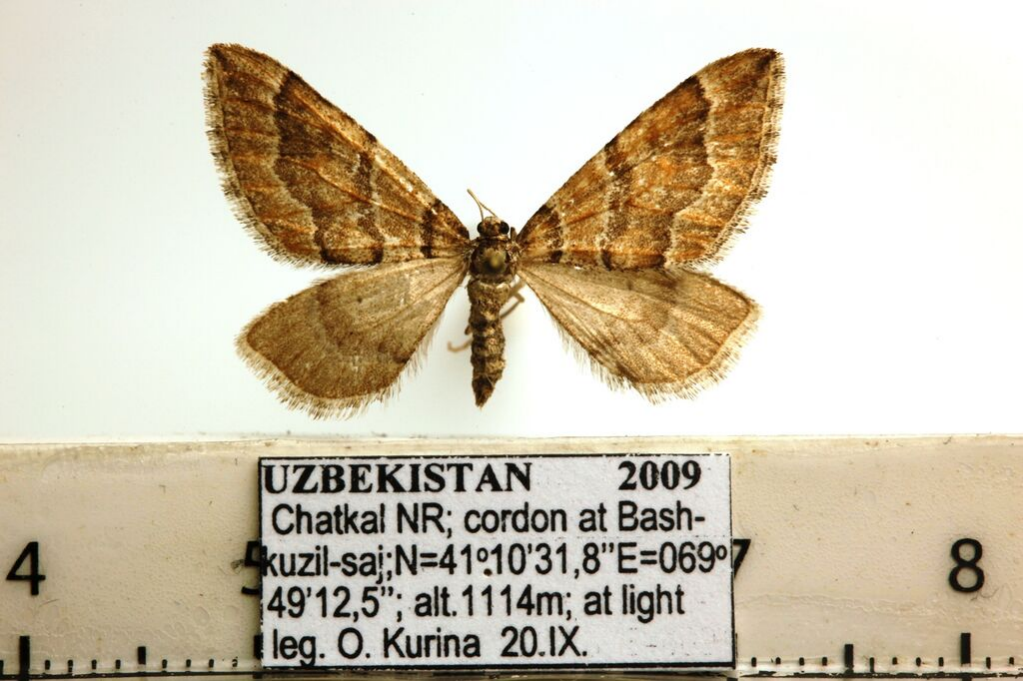
*Pljushtchiaargoi* sp. n., female paratype.

**Figure 4.**
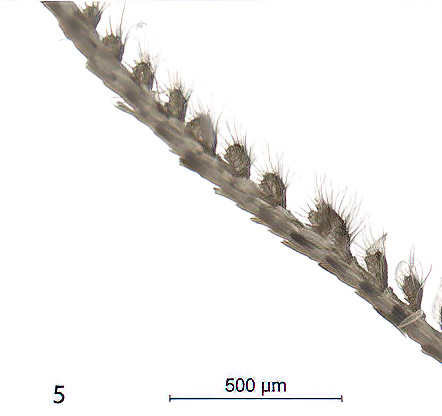
*Pljushtchiaargoi* sp. n., male antenna.

**Figure 5.**
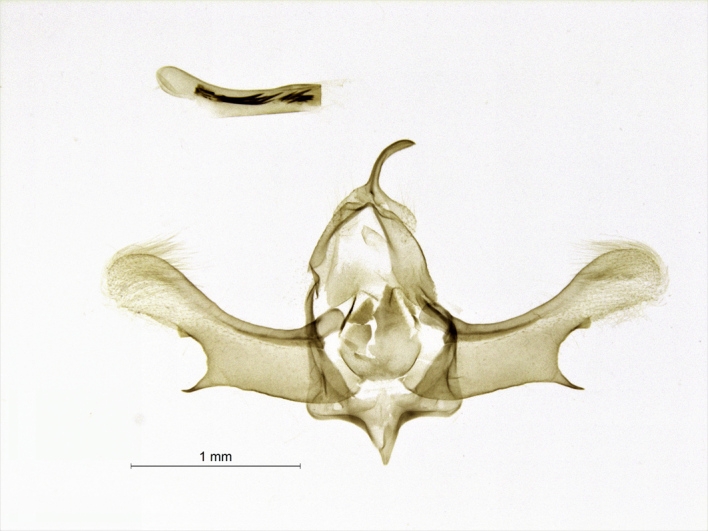
*Pljushtchiaargoi* sp. n., male genitalia and aedeagus, holotype.

**Figure 6.**
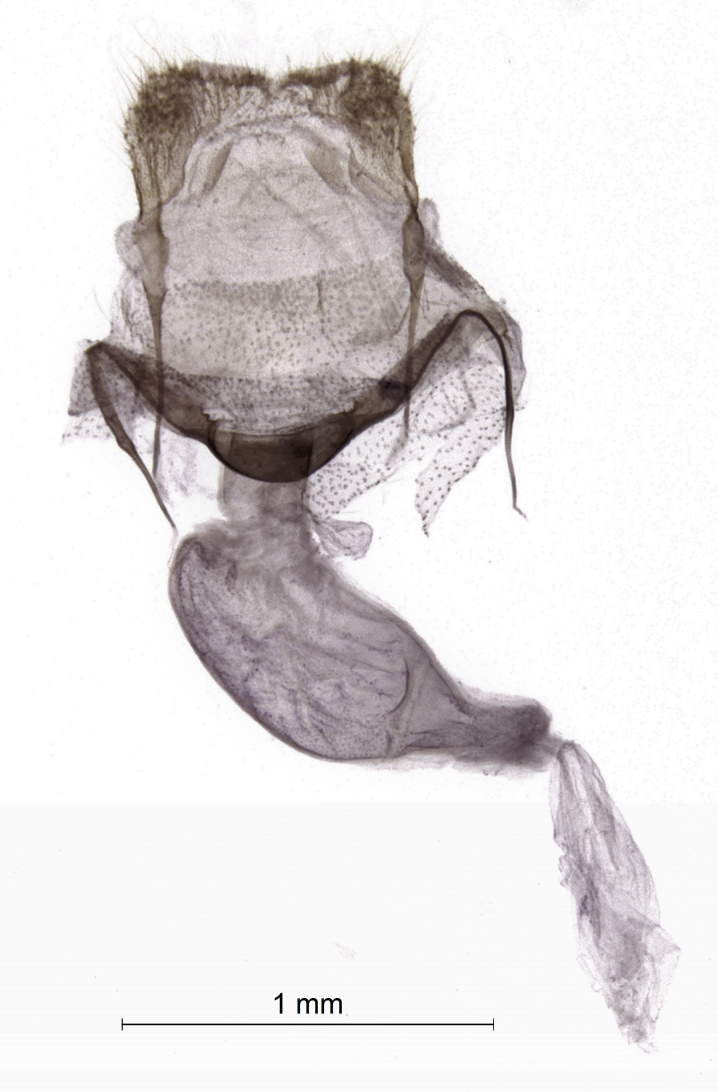
*Pljushtchiaargoi* sp. n., female genitalia, paratype.

Moths medium-sized, with 21-25 mm wingspan (Figs 2, 3). Collar and thorax greyish-brown, abdomen grey. Frons projecting about 1/3 length of eye diameter. The shape of antennae is very peculiar (Fig. 4), unipectinate or perhaps defined better as lamellate ventrally in male, the middle segments having short, flat, spoon-shaped, distally broader rounded projections which are longer than diameter of flagellum. Female antennae are saw-dentate ventrally. Forewing in male apically suffused darker, warmer dark brown in female; medial and antemedial fasciae almost straight, postmedial fascia outcurved at the cell end; hind-wing light brown-grey with a pale postmediane fascia which is edged grey on both sides; discal spot grey, uncontrasting in female, almost absent in male. Male genitalia (Fig. 5) as described for the *P.prima*, but valva simple, emarginated ventro-distally, its ventral edge with triangular projection. Labides nearly filiform. Aedeagus 1.0 mm long, the longer cornuti bundle being 0.5 mm long and reaching the base of the subapical bundle of short cornuti. Female genitalia (Fig. 6) with antrum wide, ductus bursae short, corpus bursae plain, without signum and provided with a membranous appendix. The last abdominal segment broad, apophyses posteriores as long as apophyses anteriores.

#### Diagnosis

Moths smaller than *P.prima* on average. The forewing pattern is dark brown in *P.argoi* (dark grey in *P.prima*) and forewing antemediane fascia is curved outwards on the hind margin of wing (straight in *P.prima*). The labides in *P.argoi* is nearly filiform and thinner than in *P.prima*. The longer cornuti set is 0.5 mm long and reaching the short, subapical set of cornuti in *P.argoi*, but shorter and not reaching the subapical cornuti in *P.prima* (cf. Viidalepp and Kostjuk 2005: fig. 3).

#### Etymology

The new species is dedicated to Mr. Argo Selin, son of the collector of the moths of the type series of *Pljushtshiaargoi* sp. n.

#### Distribution

Western Tien Shan, Chatkal Range.

#### Conservation

A relict of the Miocene epoch (see below), endangered by the climate change and grazing stress (Zhumanova et al. 2021). Chatkal National Reserve is mostly a pastureland, which is overgrazed despite its protected status (Sokolov et al. 1990, Borchardt et al. 2011).

#### Biology

The moth is univoltine, autumnal, confined to the lower edge of the forest tier of Chatkal Range. The local climate is characterised by the winter-spring rainfall (Aizen et al. 1997) and dry summer.

## Discussion

Our discovery of the second species of *Pljushtchia* north of the Ferghana Valley, in the Chatkal National Reserve, significantly expands the known distribution area of the so far monotypic genus. The type species—*P.prima*—has been collected together with such endemics of Hissar Range as the species of *Phthorarcha* Meyrick, *Ramiria* Viidalepp and others, which are consuming wild apple leaves. The Tajikistanian pests of fruit trees were investigated by Degtjareva (1973). The food plant association with wild apple trees (*Malussieversii*, *M.hissarica*, *M.turkestanica*) and the occurrence in relic fruit tree forest sites, suggest the possibility of co-evolution of *Pljushtchia* (and also *Phthorarcha* spp., *Ramitia* spp.) moths with wild apple trees. The origin (Wang et al. 2018), evolutionary history (Spengler 2019), phylogeny (Harris et al. 2002) and distribution of wild fruit trees has intrigued arborists, gardeners and taxonomists and are rather well studied (Yousefzadeh et al. 2019 and references therein). The divergence time of ancestors of *Malus* and *Pyrus* from the ancestral Rosales is dated as during the Eocene epoch (Wang et al. 2018). According to Wang et al. (2018), Yousefzadeh et al. (2019) and Brookfield (2000), the distribution of *Malussieversii* (the ancestor of *Malusdomestica*) out of the Ili Region, along the northern coast of Tethys Sea, to North Iran and Europe, occurred during the Miocene epoch. Since then, *Malussieversii* has radiated southwards as *Malusturkestanica* and *Malushissarica*.

It seems not usual that the same moth species inhabits mountain forests north and south of the western part of the Fergana Valley. This problem needs a special study. The fauna of Tien Shan is fragmentarily known – just the hotspots around Almaty and in the westernmost parts of the huge mountain ridge are sufficiently studied (see Viidalepp (1988) for a brief review). Gorbunov (2011) has published a review of moths of Kazakhstan, including Geometridae.

The fauna of desert, steppe and arable areas surrounds both Tien Shan and Hissar from the West: as an example, rose-feeding *Cidariafulvata* Forster reaches the 1500 m elevation in Tien Shan and 1800 m elevation on the southern flank of Hissar Mountains. The fauna of alpine meadows does not differ between these ridges as suggested by the spatial distribution of the genera *Stamnodes* Guenėe, *Grumia* Staudinger and *Nychiodes* Lederer (Viidalepp 1988). *Phthorarchaishkovi* Viidalepp is an apple tree pest in Tien Shan, whereas other species of this genus are pests in Fergana Valley and Hissar orchards (Degtjareva 1973). The green coloured moth *Chlorissaarkitensis* Viidalepp, occurs in Western Tien Shan and Degtjareva (1973) lists the vicarious *C.gigantaria* (Staudinger) (as *C.pretiosaria* Staudinger) for orchards of the Hissar. Some *Artemidora* Meyrick species (*A.maracandaria* Erschov, *A.alpherakyi* Wagner) are common in both mountain systems at 1000-3000 m elevation, while *A.metsaviiri* Viidalepp and *A.ardea* Weisert do not reach Tien Shan in their distribution. The *Artemidora* larvae are feeding on Rosacea (Degtjareva 1973). Old mountains of Tien Shan with the Hissar Range (Brookfield (2000), Allan et al. 2021), connected by the Alay Range, are discussed from the Permian epoch on the northern coast of the Tethys Sea (Sinitsyn 1962), whereas the insular Pamir and Tibet blocks are mentioned since the Triassic (Burtman 2010) and have accreted Asia later, as well as parts of China. Subtropical forests covered the mainland in the Tertiary (Wang et al. 2018). The flora and fauna of these old mountains are rich in endemic taxa compared to that of Pamir (Raduła et al. 2021), which was uplifted in the Holocene after collision of the block of Hindostan with Asia (Tong 1994, Yin 1994i). The tertiary subtropical forests are pushed from lowland to the montane belt due the Holocene climate gradual cooling and drying (Bruch and Zhilin 2007, Zhumanova et al. 2021). The recent climate change accelerates the extinction of tertiary relics of fauna and flora (Aizen et al. 1997). The westernmost Tien Shan has its own genus-level endemics as *Tshimganitia* Wehrli and *Ratsa* Viidalepp & Kostjuk, whereas the genus *Hissarica* Viidalepp seems restricted to the southern Hissar Range.

## Supplementary Material

XML Treatment for Pljushtchia

XML Treatment for Pljushtchia prima

XML Treatment for Pljushtchia argoi
